# Trunk Refugia: A Simple, Inexpensive Method for Sampling Tree Trunk Arthropods

**DOI:** 10.1093/jisesa/ieaa012

**Published:** 2020-03-18

**Authors:** Ibrahim N A Salman, Marco Ferrante, Daniella M Möller, Efrat Gavish-Regev, Yael Lubin

**Affiliations:** 1 Mitrani Department of Desert Ecology, Ben-Gurion University of the Negev, Midreshet Ben-Gurion, Israel; 2 Centre for Ecology, Evolution and Environmental Changes (cE3c), Azorean Biodiversity Group, Faculdade de Ciências Agrárias e Ambiente, Universidade dos Acores, Angra do Heroísmo, Portugal; 3 The National Natural History Collections, The Hebrew University of Jerusalem, Jerusalem, Israel

**Keywords:** arachnid, bark fauna, insect, monitoring, spider

## Abstract

Trees host a large share of the global arthropod diversity. Several methodologies have been described to sample arthropods from trees, ranging from active sampling techniques (e.g., visual searching, beating, or shaking the branches) to passive sampling devices. The majority of these collection techniques are destructive, and do not specifically target the tree trunk arthropod fauna. Here, we describe an alternative sampling method called trunk refugia (TR). TR are cylindrical shelters made of corrugated cardboard that can be secured to trees using string, and can remain exposed for varying time periods. These refugia are inexpensive, easy to use, and suitable to monitor a diverse array of insects and arachnids. Moreover, TR are nonlethal sampling tools, and allow collecting live individuals for behavioral studies or for rearing.

## Resumen

Los árboles albergan una gran parte de la diversidad global de artrópodos. Varias metodologías han sido descritas para muestrear artrópodos de árboles, desde técnicas activas (e.g., búsqueda visual, red de golpeo) hasta dispositivos de muestreo pasivo. La mayoría de estas técnicas son letales, y no están dirigidas específicamente a los artrópodos de troncos de árboles. Describimos aquí un método alternativo llamado trampas refugio (TR). Las TR son refugios hechos de cartón corrugado que se colocan en los troncos usando cuerda, y pueden permanecer expuestos por períodos variables. Estas trampas son económicas, fáciles de usar, y apropiadas para monitorear una gran variedad de insectos y arácnidos. Además, las TR son no-letales, por lo que permiten la colección de individuos vivos para estudios de comportamiento o crianza.

Trees are an essential component of almost every ecosystem around the world, and can host a large number of arthropods both in tropical (Basset et al. 2012) and temperate areas (Floren and Schmidl 2008). The importance of trees to support biodiversity is such that describing the number of arthropod species associated with a tree species is an established way to estimate global arthropod diversity (Erwin 1982, Basset et al. 1996). For arthropods, a single tree consists of several micro-habitats that can serve as refuges (Murdoch et al. 1989), overwintering sites (Balfour et al. 1975, Pekár 1999), foraging sites for herbivores that feed on the canopy (Stork et al. 1997, Ribeiro et al. 2005), as well as for predators that feed on those herbivores (Davidson et al. 2003).

Numerous methodologies have been developed to sample arthropods from trees. Several types of passive traps (e.g., light traps, water traps, flight traps, and lure traps) are frequently used as alternatives to, or in combination with, active visual searching, beating or suction sampling, and collection based on fumigation (Henderson and Southwood 2016). Most trapping tools, however, are not specific for a particular tree micro-habitat, but collect arthropods that live on trees as well as arthropods that are only active on trees sporadically. Many trapping tools have been designed to sample the tree canopy, especially when it is difficult to access, as in tropical rainforests (Basset et al. 2012). Yet, when the micro-habitat of interest is the tree trunk, fewer sampling tools are available. Tree trunk arthropods can be collected by hand, which is laborious and often impractical. Some specific tools to collect trunk arthropods exist, such as eclectors (Majer et al. 2003, Blick 2011), circular punches (Furniss 1962), ‘up and down-traps’ (Moeed and Meads 1983), ‘bottle traps’ (Pinzón and Spence 2008), and polyethylene plastic bubble wrap sheets (Isaia et al. 2006, Pinzón and Spence 2010). Most of these methods are expensive, cumbersome to operate, and lethal to the arthropods.

Trunk refugia (TR) proposed here are made of corrugated cardboard and are an effective, inexpensive, easy to use, and nonlethal tool to collect arthropods, which may be desirable for collecting live individuals (e.g., for behavioral experiments or for rearing). The spaces created by the corrugated feature of the cardboard replicate the natural crevices in the tree trunk and are used by several arthropod orders. The refugia are left in the field for days or weeks before being retrieved and thus sample arthropod activity as well as abundance. Strips of corrugated cardboard rolled around tree branches (Isaia et al. 2006), inserted within the trunk (Fye 1985), or secured around the tree trunk (Pekár 1999, Korenko and Pekár 2010), have been used to collect arthropods, spiders in particular, from urban areas and orchards.

Here, we describe a new version of these corrugated cardboard sampling tools and demonstrate their use to collect a great diversity of arthropod orders from three different ecosystems: a hyper-arid desert, pomegranate orchards, and an oak woodland. To our knowledge, this is the only time-efficient method to sample live arthropods from the tree trunk, and therefore, we could not compare it with others. We suggest this method for ecological monitoring, biodiversity assessment, and the collection of live specimens.

## Methods

### Trunk Refugia

**Fig. 1.**
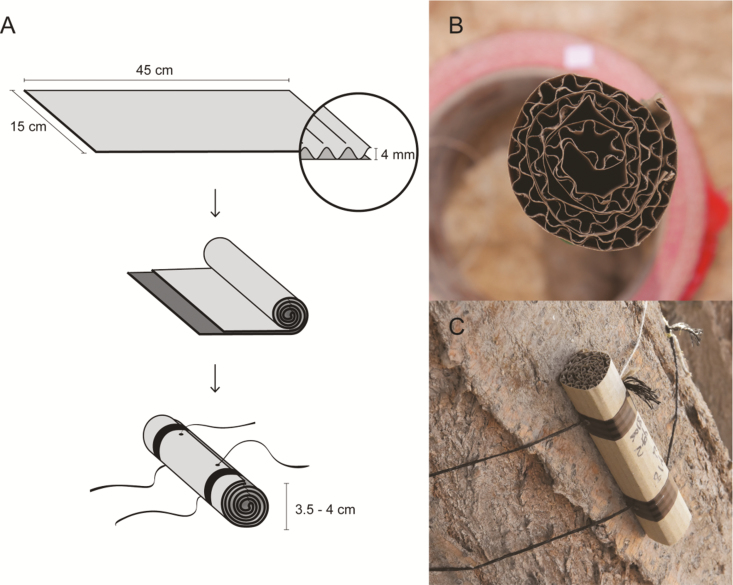
Trunk refugia: (A) preparation, (B) top view, (C) TR attached to the tree trunk. Figure 1B: Photo taken by Jakob Guebel.

Each TR is made of a rectangular piece of single-face corrugated cardboard (see specific experiments for details on the size) folded longitudinally, approximately in half and rolled up to form a cylinder (Fig. 1A). The cylinder must not be rolled up too tightly to allow arthropods to enter the refugia (Fig. 1B), and it can be secured by wrapping adhesive tape around each end of the cardboard cylinder. Using a drill or scissors, two small holes can be made crosswise through the cylinder, one near each end. A string is inserted through each hole, which serves to attach the TR to the tree trunk (Fig. 1C).

The TR can be secured tightly to the trunk at the desired height, making sure that it is in contact with the trunk to allow arthropods to enter. After being exposed for a set time, the TR can be easily collected by cutting the strings and placing it in a plastic bag. Collected TR can be unrolled and unfolded inside a tub or bucket to prevent the escape of fast-moving arthropods when opening it. After the arthropods on the outer surfaces of the cardboard are collected, the two layers of the material can be easily split apart by applying a small amount of water to both sides of the unfolded cardboard with a damp sponge to loosen the glue, which allows for the collection of the arthropods hiding between the layers of the cardboard.

A pilot study was conducted to test different TR modifications and the technique was applied in several agricultural and native landscapes in Israel. Unless otherwise specified, all arthropods were identified to order except for spiders, which were classified into families.

### Testing Different TR Modifications

We tested three different TR modifications on pomegranate trees (*Punica granatum* L.) in an organic orchard at Kibbutz Ne’ot Semadar (30°03′05′′ N, 35°02′10′′ E), in the Negev desert, southern Israel during the summer 2016. The trees all had multiple trunks. Twenty-six pomegranate trees were selected, and on each tree, three different TR were placed, all 15 cm long: 1) narrow TR (diameter ~ 3.5 cm), 2) covered narrow TR (diameter ~ 3.5 cm with the upper end covered with plastic wrap) and, 3) wide TR (diameter ~ 4 cm), for a total of 78 traps. All TR were deployed in a vertical orientation with respect to the trunk, on three separate trunks of each tree, at 1–1.5 m from the ground and tied to the tree trunk with string. After 35 d, the TR were collected and opened in the lab to collect all arthropods. Arthropods were identified and counted, and their abundance at the order level in the three TR modifications were compared using the Wilcoxon Signed-rank test.

### *Vachellia* Trees: ‘Avrona Nature Reserve

Arthropod diversity in a hyper-arid desert ecosystem was assessed using TR (15 cm long and 3.5 cm in diameter) in ‘Avrona Nature Reserve (29°41′22′′ N, 34°59′25′′ E), in the ‘Arava Rift Valley, southern Israel. Between February and September 2019, 30 trees (20 *Vachellia* [formerly *Acacia*] *tortilis* (Forssk.) Hayne and 10 *Vachellia raddiana* (Savi) were sampled every month or second month. On each *Vachellia* tree, two TR were placed approximately 1 m above ground and were left exposed for about a month (mean = 31 d, SD = 5 d), after which TR were collected and then opened in the lab to collect arthropods and identify them to order or family level.

### Pomegranate Orchards Along a Precipitation Gradient

Twelve organic pomegranate orchards were selected along the latitudinal climatic gradient of Israel during the summer of 2015. For details and locations of the study sites, see Salman et al. (2019). In each orchard, we placed one TR (15 cm long and 3.5 cm in diameter) per tree on six trees at the orchard center and on six trees at the orchard edge (12 traps per orchard), for a total of 144 traps. TR were deployed twice, once in June and again in August, and were collected each time after approximately 1 mo (mean = 30 d, SD = 5 d).

### Oak Trees: Jerusalem Botanical Gardens

As part of an undergraduate class exercise, TR (15 cm long and 3.5 cm in diameter) were placed on six oak trees (*Quercus* sp.) in the Jerusalem Botanical Gardens, Giva’at Ram (31°46′N, 35°11′E), Israel, for 7 d during April 2018. On each oak tree, two TR were placed: one at 20–40 cm and another at 1.2–1.7 m above the ground, totaling 12 TR.

## Results

### Testing Different TR Modifications

We collected 534 arthropods from 12 orders (Table 1). Narrow TR collected more arthropods (239 individuals, 11 orders) than covered narrow TR (153 individuals, 9 orders) and wide TR (142 individuals, 10 orders). The main arthropod orders collected in the narrow TR were Blattaria (33.5%), Araneae (29.3%), and Hymenoptera (15.9%). In the covered narrow TR, the most common order was Araneae (56.2%), followed by Acari (11.8%), and Hymenoptera (9.8%). In the wide TR, the most common orders were Hymenoptera (39.4%), Acari, and Araneae (both shared 15.5%). Salticidae was the most common spider family in the wide TR (27.3%) and in the narrow TR (71.4%). We were unable to identify the majority of the spiders (91.9%) in the covered narrow TR because they were juveniles (Table 2). There were no significant differences among the total numbers of arthropods of the different orders captured in the different TR modifications (Wilcoxon Signed-rank tests, narrow: wide, Z = 0.56, *P* = 0.59; narrow: covered, Z = 0.63, *P* = 0.55; covered: wide, Z = −0.94, *P* = 0.37).

**Table 1.** List of arthropod orders and percentage of total captures by the three different types of TR in Ne’ot Semadar, Israel

		Percentage (%)	
Arthropod order	TR-wide	TR-covered-narrow	TR-narrow
Total number	142	153	239
Araneae	15.49	56.21	29.29
Acari	15.49	11.76	5.02
Blattaria	6.34	1.96	33.47
Coleoptera	4.23	2.61	4.60
Hemiptera	0.00	1.31	1.26
Hymenoptera	39.44	9.80	15.90
Lepidoptera	0.70	0.00	0.00
Orthoptera	0.70	0.00	1.67
Psocoptera	5.63	3.27	1.67
Scorpiones	0.00	0.00	0.42
Solifugae	4.23	1.96	3.35
Thysanoptera	6.34	6.54	2.93
Unidentified	1.41	4.58	0.42

**Table 2.** List of spider families and percentage of captures by the different types of TR in Ne’ot Semadar, Israel

		Percentage (%)	
Spider family	TR-wide	TR covered-narrow	TR-narrow
Total number	22	86	70
Gnaphosidae	4.55	0	1.43
Linyphiidae	4.55	0	1.43
Oonopidae	4.55	0	0
Philodromidae	4.55	2.33	2.86
Salticidae	27.27	2.33	71.43
Scytodidae	9.09	1.16	1.43
Theridiidae	0	0	1.43
Thomisidae	0	1.16	2.86
Zodariidae	9.09	1.16	0
Unidentified	36.36	91.86	17.14

### *Vachellia* Trees: ‘Avrona Nature Reserve

**Fig. 2.**
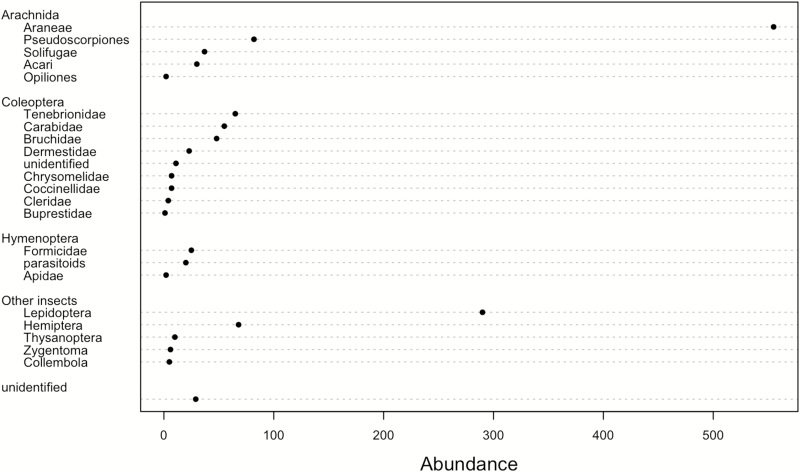
Arthropods collected using trunk refugia in ‘Avrona Nature Reserve (southern Israel) between February and September 2019.

We collected 1,320 arthropods from 13 orders. The most abundant taxa were Araneae (40.2%) and other arachnids (10.9%), followed by Lepidoptera (21.0%, of which >95% were caterpillars and 5% pupae), Coleoptera (15.2%), and other insect orders combined (Hymenoptera, Hemiptera, Thysanoptera, Zygentoma, Collembola, unidentified; 12.7%; Fig. 2).

### Pomegranate Orchards Along a Precipitation Gradient

**Fig. 3.**
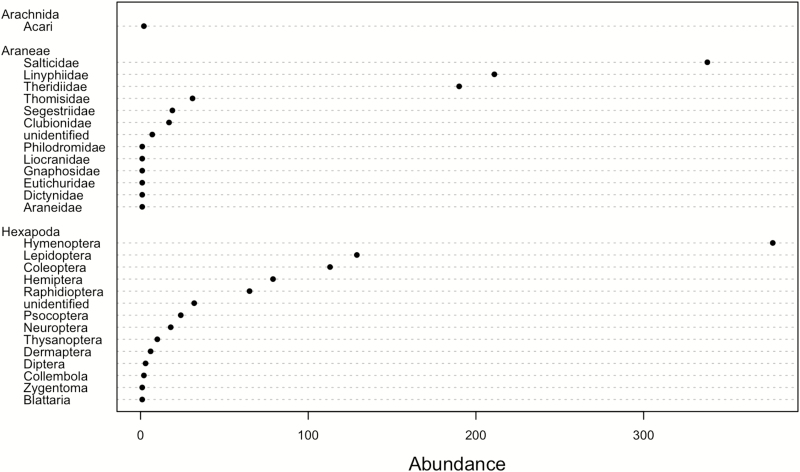
Abundances of arthropods collected in pomegranate orchards using trunk refugia during the summer 2016.

We collected 1,681 arthropods from 15 orders (Fig. 3). The most abundant taxa were Araneae (48.7%) followed by Hymenoptera (22.4%), Lepidoptera (mostly caterpillars, 7.7%), Coleoptera (6.7%), Hemiptera (4.7%), and Raphidoptera (3.9%, all of them were larvae). Other orders included Blattaria, Collembola, Dermaptera, Diptera, Neuroptera, Psocoptera, Thysanoptera, Zygentoma, and unidentified arthropods (altogether comprised 5.9%).

### Oak Trees: Jerusalem Botanical Gardens

We collected 133 Dermaptera and 12 Araneae (four Salticidae, three Gnaphosidae, three Linyphiidae, and two Theridiidae).

## Discussion

We showed how TR can be used to collect several arthropod orders (up to 18 in our combined experiments), some of which were obtained in large numbers. Our test of TR modifications showed that spiders were relatively more abundant in the narrow traps, and these were used in the pomegranate orchard and native trees studies, which focused mainly on arachnids. The particularly high abundance of spiders is not surprising, as arachnids are the main arthropod predators in arid ecosystems (Polis 1991, Ayal 2007), and similar refugia devices have been used to target specifically spiders (Pekár 1999, Isaia et al. 2006, Korenko and Pekár 2010). Nevertheless, this monitoring tool is as effective for insects as for arachnids, even though its use among entomologists remains undocumented to our knowledge. In pomegranate orchards in the semi-arid environments, Hymenoptera and Blattaria were particularly abundant, while on *Vachellia* trees in a hyper-arid desert ecosystem, Lepidoptera and Coleoptera from several families were the main taxa. Very large numbers of trunk-dwelling arthropods may use the TR even when the refugia are exposed only over a few days; for example, TR placed on oak trees in a botanical garden collected a large number of earwigs in 1 wk. Moreover, these results demonstrated that TR are not only a useful tool for monitoring arthropod diversity, but also for collecting live individuals. This advantage makes TR suitable for obtaining individuals to rear for use in behavioral or physiological experiments. It also enables one to release nontarget groups, which should be seriously considered given the ongoing anthropogenically induced mass decline in arthropod numbers (Drinkwater et al. 2019).

Determining the reasons why the different members of the arthropod community of tree trunks use these traps as refugia was beyond the scope of our study. Yet, we found evidence that arthropods used them for reproduction (e.g., spider egg sacs and wasp and bee nests), development (e.g., spider exuviae and immature stages of Coleoptera, Hemiptera, and Lepidoptera), and as hunting sites (e.g., ant and beetle fragments). Likely, some taxa such as Lepidoptera, Hemiptera, and Bruchidae used the TR also to avoid predators and escape extreme climatic conditions, particularly in desert ecosystems.

### Benefits, Limitations, and Recommendations

#### TR design

Numerous modifications of the design described here are possible. Our TR were constructed of cardboard, a design suitable for a dry climate but perhaps not for a wet environment. Materials that can withstand rain and fog and thus, prevent the TR from collapsing should be tested, such as 100% recyclable polypropylene. From a monitoring perspective, a larger (longer, wider) TR may be more effective in capturing more species and more individuals, and using thicker cardboard with larger internal spaces may collect larger arthropods. TR to be placed on small branches could be long and narrow in shape.

#### TR placement and exposure duration

TR are easily adapted to address specific questions. For example, information on seasonal changes in the relative abundance of arthropods can be obtained by repeated exposure of TR over the year. Information on the timing of reproduction can be obtained for those species that use these shelters as breeding sites. Different parts of the tree (different heights, trunk vs branches) are easily compared, as are different tree species.

TR may be deployed for any convenient amount of time, but we recommend to avoid excessively short periods to give enough time for arthropods to discover and start using the refugia, as well as excessively long periods (e.g., more than a month) to reduce the risk of the refugia being damaged by weather conditions.

#### Cost and benefit

Although each trap can be used only once, they are inexpensive and easy to construct. Large rolls of corrugated cardboard can be purchased commercially, and the only additional materials needed are string and tape. This allows one to conduct numerous arthropod sampling events at low cost. Note, however, that the method requires two field visits for each sampling event—one to place the TR and another to retrieve them.

In summary, we suggest that TR constitute a versatile, inexpensive sampling tool for tree trunk-dwelling arthropods, a habitat that is consistently under-represented in entomological studies in comparison with ground and canopy habitats. We note that an additional, potential use of TR may be to augment populations of natural enemies (e.g., spiders and predatory hymenopterans) on orchard trees by providing them with suitable shelter.

## Notes

The first two authors contributed equally to this manuscript.
